# An optimized method for RNA extraction from the polyurethane oligomer degrading strain *Pseudomonas capeferrum* TDA1 growing on aromatic substrates such as phenol and 2,4-diaminotoluene

**DOI:** 10.1371/journal.pone.0260002

**Published:** 2021-11-15

**Authors:** María José Cárdenas Espinosa, Tabea Schmidgall, Georg Wagner, Uwe Kappelmeyer, Stephan Schreiber, Hermann J. Heipieper, Christian Eberlein

**Affiliations:** 1 Department of Environmental Biotechnology, Helmholtz Centre for Environmental Research - UFZ, Leipzig, Germany; 2 Department Molecular Systems Biology, Helmholtz Centre for Environmental Research - UFZ, Leipzig, Germany; Universidade Estadual de Ponta Grossa, BRAZIL

## Abstract

Bacterial degradation of xenobiotic compounds is an intense field of research already for decades. Lately, this research is complemented by downstream applications including Next Generation Sequencing (NGS), RT-PCR, qPCR, and RNA-seq. For most of these molecular applications, high-quality RNA is a fundamental necessity. However, during the degradation of aromatic substrates, phenolic or polyphenolic compounds such as polycatechols are formed and interact irreversibly with nucleic acids, making RNA extraction from these sources a major challenge. Therefore, we established a method for total RNA extraction from the aromatic degrading *Pseudomonas capeferrum* TDA1 based on RNAzol^®^ RT, glycogen and a final cleaning step. It yields a high-quality RNA from cells grown on TDA1 and on phenol compared to standard assays conducted in the study. To our knowledge, this is the first report tackling the problem of polyphenolic compound interference with total RNA isolation in bacteria. It might be considered as a guideline to improve total RNA extraction from other bacterial species.

## Introduction

During several decades, various microorganisms have evolved metabolic pathways to degrade environmental pollutants derived from anthropogenic activities (e.g. agriculture, solid waste, untreated industrial effluents, oil and solvent industry, etc.) which are present in different habitats [1]. In nature, aromatic compounds belong to the most persistent and hazardous pollutants, causing deleterious effects on human and animal health [2,3]. Their biodegradation has been intensely studied, however the removal of organic compounds has been focused on the role of bacteria due to their quick adaptation, metabolic versatility and genetic plasticity allowing them to use aromatic substrates as their sole carbon and energy source [4,5]. Hundreds of bacterial species of several phylogenetic origins have been identified as being capable to aerobically degrade all different kinds of aromatic compounds. Among all these, *Pseudomonas* species are by far the best studied ones because of their unique properties to degrade and tolerate a wide variety of xenobiotic compounds [6,7].

The mayor challenge in bacterial degradation is to overcome the resonance energy that stabilizes the aromatic ring [8,9]. To do so, aerobic bacteria rely on the addition of either one or two atoms of molecular oxygen by mono or dioxygenases. They transform aromatic compounds into central intermediates of aromatics degradation such as catechol, protocatechuate and gentisate [10]. Then, ring-cleaving dioxygenases catalyze ring fission via the *ortho*- or *meta*-cleavage pathway. During *ortho*-cleavage pathway, the aromatic ring fission occurs between two hydroxyl groups while during *meta*-cleavage this is done between one hydroxylated carbon and other adjacent non-hydroxylated carbon. Both ways are catalyzed by intradiol and extradiol dioxygenases, respectively, using Fe^+3^ and Fe^+2^ at the active site [2,11,12]. Finally, ring cleavage products are transformed into aliphatic molecules that can be channeled to the central metabolism. Some central intermediates of the biodegradation of aromatics such as phenols and catechols can be easily oxidized to yield the corresponding quinones. This reaction is regulated by the activity of enzymes known as polyphenol oxidases (PPOs) which are the principal basis of the browning reactions in plant tissues and extracts [13–16]. Such oxidases were also observed in *Pseudomonas* and other bacterial species [17]. Several authors have suggested that enzymatic oxidation of phenolic compounds and the presence of polysaccharides and other secondary metabolites represent a major problem in molecular studies [18–21]. A modified and improved RNA isolated protocol have been described in a bacterial culture containing pyrene as a carbon source [22].

Nucleic acid isolation is regularly the starting point for all downstream applications. However, isolation of intact RNA can be a challenge due to several factors including hydrolysis susceptibility, enzymatic and heat degradation [23]. To overcome these problems, reliable extraction methods such as commercial RNA extraction kits and organic solvents yield high quality RNA from different types of samples including cell lines, plant and mammalian tissues, bacteria, virus, etc. Nevertheless, phenolic compounds, polysaccharides, proteins and other secondary metabolites interfere with nucleic acids tend to co-precipitate or degrade RNA, restricting its yield and quality [13,23–27].

In many follow up applications including cDNA library construction, gene expression studies and next generation sequencing, the reproducibility and validity of the data depend on the quality of the RNA extracted [26]. In addition, the accurate assessment of RNA integrity and the correct quantification are key elements for further molecular analysis.

In this study, total RNA extraction was conducted for the *Pseudomonas capeferrum* TDA1 growing on three different carbon sources including phenol, succinate and 2,4-diaminotoluene (2,4-TDA); an aromatic diamine and precursor for the production of polyurethane. In previous reports, this compound was degraded by *Pseudomonas capeferrum* TDA1 and a preliminary degradation pathway was suggested [28,29]. Regardless of the proposed pathway, mono- and dioxygenases are involved for sure, leading to polycatecholic/phenolic intermediates. Those are likely to be subjected to the activity of oxidases present in the strain’s genome and responsible for the formation of polyphenolic compounds observed as dark precipitation during growth on 2,4-TDA. In order to obtain high quality RNA, commercial kits and conventional methods were tested but all of them failed for the cells grown in 2,4-TDA media, most likely due to the presence of polyphenolic compounds that could interfere with the RNA [30–34]. In order to solve this problem, a simple and effective RNA extraction method from bacterial cultures grown on 2,4-TDA was developed. This procedure uses a mixture of guanidine thiocyanate and phenol in a monophasic solution, which is a frequent protocol for some varieties of biological samples considered as a “challenge” due to several factors. It provides purified total RNA suitable for RT-PCR, qPCR and cDNA libraries.

## Materials and methods

### Bacterial strain and growth conditions

Prior to the experiment, *Pseudomonas capeferrum* TDA1 was cultivated in Hartman’s mineral salts medium [28] and succinate (4 g/L) as carbon source at 30°C and 150 rpm overnight. Afterwards, two milliliters of each culture were centrifuged (7 minutes at 18,000 g) and the resulting cell pellets were washed with KNO_3_ (10 mM) while the supernatant was discarded.

The pellets were added to mineral media containing only one carbon source (4 g/L succinate, 2 mM 2,4-TDA or 2 mM phenol) and incubated for 8 hours (succinate) and 7 days (2,4-TDA) until they reached the exponential phase (OD_560_ = ~0.8). The cells grown on phenol were harvested after 5 days and added to a fresh medium, reaching the exponential phase 4 days later. After that, two milliliters of the culture of every single media were centrifuged (3 minutes at 18,000 g), re-suspended in RNA Later (Sigma-Aldrich, St. Louis, MO, USA) and stored at -80°C.

### RNA isolation

The following standard laboratory kits and methods were tested for RNA extraction from *Pseudomonas capeferrum* growing on succinate, phenol and 2,4-TDA following the manufacturer’s instructions: RNeasy, RNeasy PowerPlant (Qiagen, Düsseldorf, Germany), peqGOLD TriFast (VWR, Leuven, Belgium) and phenol chloroform protocol [35]. Additionally, the improved protocol was applied for all three carbon sources mentioned. This was done by using RNAzol^®^ RT (Sigma-Aldrich, St. Louis, USA) with the following modifications: Initially, the cell solution was centrifuged (5 minutes at 20,000 g) to collect cells and discard the supernatant. 0.5 milliliters of RNAzol^®^ RT were added to the pellets and re-suspended in the reagent. Each solution was transferred to the lysing matrix B tubes and homogenized using the FastPrep-24 (MP Biomedicals, Inc) during 35 seconds at 6.5 m/s. After homogenization, the samples were transferred to 1.5 mL micro-centrifuge tubes and 0.2 mL of RNase-free water were added for DNA, protein, and polysaccharide precipitation according to the protocol [36].

**Fig 1:**
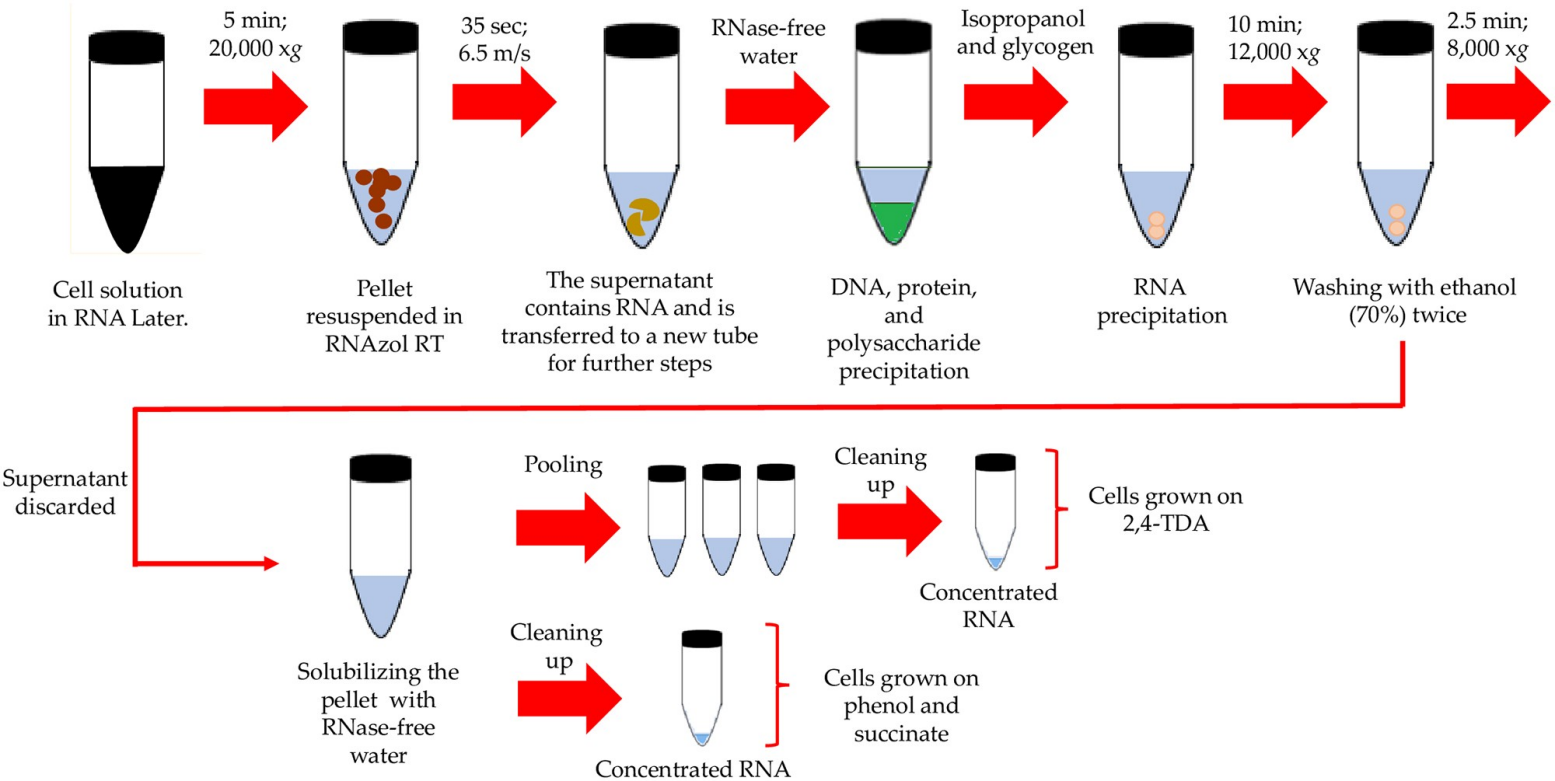
Optimized RNA extraction method from *Pseudomonas capeferrum* TDA1.

Afterwards, the supernatant was transferred to a new 1.5 mL low binding micro-centrifuge tube with an equal volume of isopropanol and 1 μL of glycogen (molecular biology grade, Thermo Fisher, Waltham, United States). The samples were incubated at −80°C for 40 minutes and centrifuged at 12,000 g for 10 minutes at room temperature. The RNA pellets were washed twice with 0.4 mL of 70% ethanol (v/v) and centrifuged at 8,000 *g* during 2.5 minutes at room temperature. The supernatant was removed carefully and the pellets were solubilized by adding RNase free water (45 μL) (Fig 1). Finally, the samples were cleaned up using the RNA Clean & Concentrator^™^-5 kit (Zymo Research, California, USA) following the protocol suggested by the manufacturer for total RNA extraction. Due to the low RNA concentration (2.5–3.0 ng/μL) yielded from 2,4-TDA samples, a pooling step (2 or 3 samples per pool) was added to the protocol before the cleaning up process.

### RNA quantification and RIN determination

Total RNA was quantified using a fluorescent RNA-binding dye Qubit Fluorometer (Thermo Fisher, Waltham, United States) according to the manufacturer’s instructions. A_260_/_280_ and A_260_/_230_ values for RNA samples were measured using Nanodrop ND-1000 Spectrophotometer (Peqlab, Erlangen, Germany). The quantification of RNA was done in triplicates. After RNA concentrations in the samples were analyzed, RNA integrity was determined in 1 μl of total RNA using the RNA Nano (succinate and phenol) and Pico chips (2,4-TDA) assays and Agilent 2100 Bioanalyzer (Agilent Technologies Inc., Santa Clara, United States) in accordance with the manufacturer’s protocols. Samples with an integrity number (RIN) above 7.0 were selected for further downstream applications.

## Results and discussion

### Yield and quality of total RNA isolated from *P*. *capeferrum* TDA1

Routine molecular applications such as RT-PCR, NGS, RNA-seq require RNA with high purity and integrity [23,26]. At present, several methods and kits are available for extracting RNA from samples rich in polysaccharides, phenols and other secondary metabolites but they are mostly applied to plants tissues, leaves and woody species [20,21,23,26,33,37,38]. For this particular reason, five RNA isolation methods were compared in order to obtain high-quality RNA from *Pseudomonas capeferrum* TDA1 growing on aromatic compounds (phenol and 2,4-TDA) and succinate.

First, two commercial kits (RNeasy and RNeasy power plant kit, Qiagen) based on spin columns following the manufacturer’s recommendations yielded low RNA quantity for phenol (ranged from 5.3 to 18.9 ng/μL) and 2,4-TDA samples (ranged from 2.0 to 2.1 ng/μL), compared to RNA isolated from cells grown on succinate that showed higher concentrations for both protocols.

In addition, one method based on phenol/chloroform [35] and another containing guanidium-thiocyanate-phenol and chloroform (TriFast, VWR) were tested and the RNA yields were the lowest for cell samples grown on the aromatic compounds among all assays conducted. Finally, the modified RNAzol RT method achieved high RNA concentrations for *P*. *capeferrum* TDA1 grown on succinate, phenol and 2,4-TDA (Table 1).

**Table 1:** Total RNA quantity (ng/μL) and purity (A_260_/A_280_ and A_260_/A_230_) for different RNA isolation methods applied on cells from *Pseudomonas capeferrum* TDA1 growing on different carbon sources (succinate, phenol and 2,4-TDA). Values represent mean ± SD.

Method	Concentration (ng/μL)			A_260_/A_280_			A_260_/A_230_		
	Succinate	Phenol	TDA	Succinate	Phenol	TDA	Succinate	Phenol	TDA
**RNeasy** ^1^	67.5±14.17	5.3±1.77	2.0±1.74	1.98±0.19	1.60±0.12	2.71±1.44	2.02±0.13	0.33±0.18	0.2±0.13
**RNeasy P.Plant** ^1^	48.7±12.35	18.9±3.02	2.1±0.25	2.25±0.06	2.06±0.09	1.30±0.46	2.12±0.05	2.02±0.18	2.48±1.58
**Phenol/Chloroform**	54.0±25.07	37.3±25.66	0.3±0.14	1.67±0.15	1.20±0.57	LOD	1.63±0.52	0.81±0.62	LOD
**Trifast** ^2^	102.7±9.38	88.2±1.17	1.1±0.71	1.66±0.37	1.85±0.07	LOD	2.17±0.08	1.65±0.28	LOD
**Modified RNAzol RT** ^3^	130.0±32.07	73.6±8.80	5.3±0.16	2.11±0.02	2.10±0.11	2.02±0.16	2.32±0.07	2.10±0.27	1.95±0.11

^1^Column purification;

^2^ Guanidium thiocyanate, phenol and chloroform;

^3^ Guanidine thiocyanate and phenol. LOD: below limit of detection.

A_260_/A_280_ and A_260_/A_230_ ratios are often used for providing a rough indication of purity. A ratio of 2.0–2.2 is generally accepted as pure RNA [26,39,40]. Table 1 shows the A_260_/A_280_ ratio for the five protocols demonstrating that RNeasy kit (1.98 ± 0.19; succinate), RNeasy power plant kit (2.25 ± 0.06; succinate, 2.06 ± 0.09; phenol) and modified RNAzol RT (2.11 ± 0.02; succinate, 2.10 ± 0.11; phenol, 2.02 ± 0.16; 2,4-TDA) method were effective inhibiting protein and phenol contamination [39,40].

The A_260_/A_230_ ratio is a sensitive indicator of contaminants such as: guanidine thiocyanate (GTC), guanidine hydrochloride (GuHCl), EDTA, polysaccharides and other secondary metabolites. The RNeasy kit (2.02 ± 0.13; succinate), RNeasy power plant kit (2.12 ± 0.05; succinate, 2.02 ± 0.18; phenol) and modified RNAzol RT (2.32 ± 0.07; succinate, 2.10 ± 0.27; phenol, 1.95 ± 0.11; 2,4-TDA) showed values that correspond to high RNA purity (Table 1). On the other hand, phenol/chloroform and TriFast methods revealed lower A_260_/A_280_ (≤ 1.85) and A_260_/A_230_ (≤ 1.65) ratios for succinate and phenol samples, which indicates organic contamination that compromises the RNA quality [23,26,38,41,42]. For these two methods, the ratios could not be measured for 2,4-TDA because the RNA concentration was below the detection limit [43].

RNA isolation of cells grown on 2,4-TDA using spin columns presented A_260_/A_280_ and A_260_/A_230_ ratios out of the acceptable range, suggesting possible problems in the extraction due to the presence of polyphenolics, polysaccharides and secondary metabolites that precipitated with the nucleic acids [26,42].

These results are consistent with previous reports which demonstrated that commercial kits using spin columns are not suitable for RNA extraction from plants rich in polysaccharides and polyphenols. Phenolic substances reduce the efficiency of the column and can bind irreversibly to proteins and nucleic acids, leading to degradation and subsequent low-quality RNA [23,26,44]. Therefore, the low RNA concentration yielded by commercial kits has been proven previously in several plant tissues, seeds, roots and woody perennials with high content of polysaccharides and polyphenols [23,25–27,42,45].

However, not all RNA yields from cells grown on aromatic compounds had the same result. In the case of 2,4-TDA, the poor RNA yield and quality demonstrated protein and organic contamination. During incubation, a browning effect in the media was observed, which suggests the presence of PPO enzymes catalysing the oxidation of diphenols to quinones [28,46] that can irreversibly bind to the RNA and interfere with the extraction process and downstream applications [18,23,31]. Studying the annotated genome of *Pseudomonas capeferrum* TDA1 reveals the presence of the gene *yfiH* encoding for a polyphenol oxidoreductase laccase (EC 1.10.3.2) (data not showed). This enzyme oxidizes a broad range of phenolic and non-phenolic compounds and has been isolated from several *Pseudomonas* species [47]. In this study, the browning effect was not visible in phenol samples probably due to the hydroxylation of monophenols to diphenols that produces colourless intermediates [46,48]. Previous work also demonstrated that high-quality total RNA could be isolated from bacterial strains grown on phenol and benzoate using commercial kits [49–51], however not all the metabolic pathways related to bio-degradation of aromatic compounds have been identified and many intermediates as well as secondary metabolites are still unknown.

Methods (phenol/chloroform and TriFast) based on guanidinium thiocyanate–phenol–chloroform were less efficient for the isolation of RNA from *P*. *capeferrum* TDA1. RNA extracted from cells grown on phenol presented low A_260_/A_280_ and A_260_/_230_ ratios (Table 1) for both protocols. These results are consistent with previous reports that suggested that chloroform can affect the isolation and quantification of the RNA [23,35]. Thus, polysaccharides can co-precipitate with RNA during the phenol/chloroform extraction steps [25]. Despite of these drawbacks, some modified protocols have been tested in plant leaves [52], seeds [37] and seedlings [53] obtaining high-quality RNA.

In the case of 2,4-TDA samples, the phenolic compounds and secondary metabolites in the oxidized form could interfere with the RNA yield. Negligible quantities of RNA extracted from plant tissues was reported earlier using a reagent based on guanidinium thiocyanate [54]. Also, it has been demonstrated that this substance participates in the precipitation of considerable amount proteins with the nucleic acids, reducing the RNA isolation efficiency [23,55].

**Fig 2:**
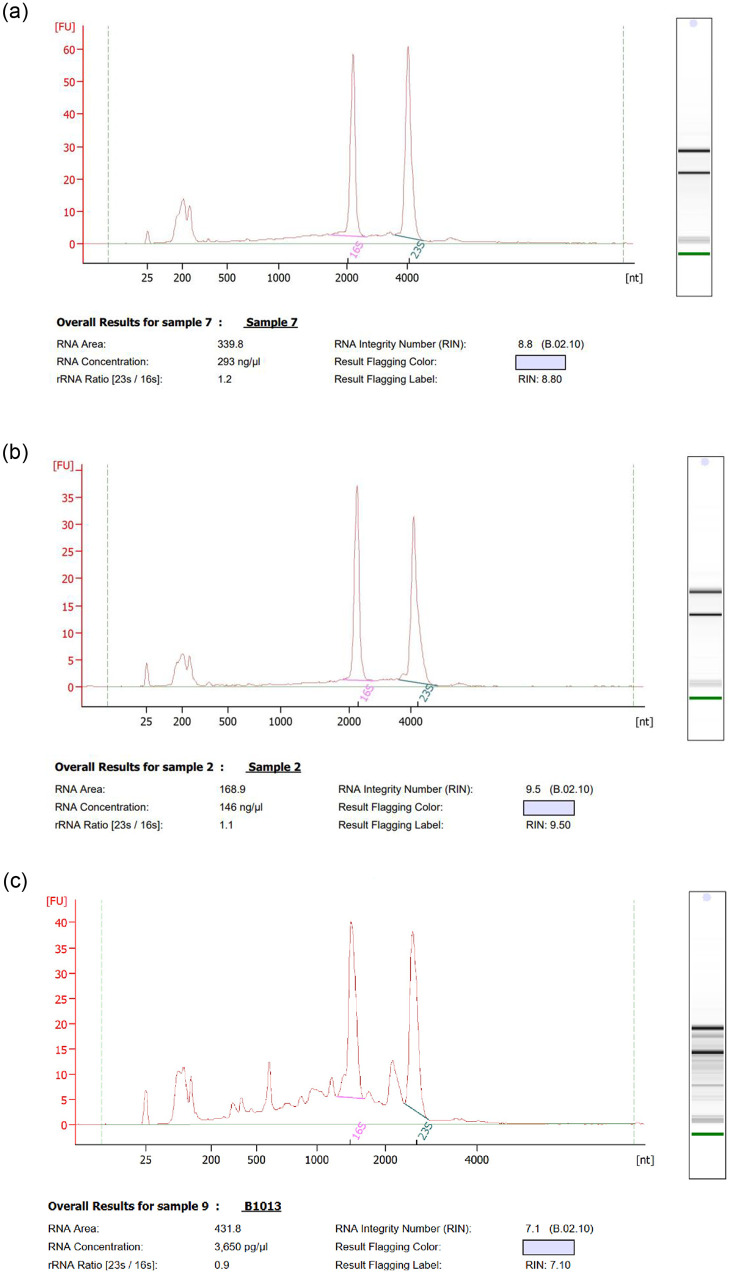
Bioanalyzer results. Electropherograms of total RNA extracted from *Pseudomonas capeferrum* TDA1 grown on: A) phenol (with TriFast method), B) phenol (with modified RNAzol RT method) and C) 2,4-TDA (with modified RNAzol RT method). The main peaks correspond to ribosomal RNA (16S and 23S).

Further analysis of RNA integrity using an Agilent 2100 Bioanalyzer showed RIN values ≥ 8.50 (Fig 2A–2C) for RNA from cells grown on phenol (TriFast and modified RNAzol RT) and for 2,4-TDA (modified RNAzol RT) indicating no degradation of RNA. Generally, RNA with a RIN value above 7.0 is suitable to ensure sequencing quality [42]. The three remaining methods revealed RIN values below 5.50.

On the other hand, RNA isolation from succinate samples (RNeasy and RNeasy power plant kit, TriFast and modified RNAzol RT) revealed RIN values ≥ 7.90, which confirms the complexity of high-quality RNA extraction from cells grown on aromatic compounds. In contrast, the phenol/chloroform protocol obtained low RIN values (≤ 5.20) for all the samples.

RNA samples (succinate and phenol) extracted with the TriFast method exhibited A_260_/_280_ and A_260_/_230_ ratios out of the acceptable range for high-quality RNA (lower than 1.8 or over 2.0) and a RIN over 8. This finding is compatible with other studies which propose that RNA purity and RNA integrity are unrelated and there is no significant correlation between them [56,57]. However, RNA quality control (integrity and purity) is critical and must be assessed independently in order to assure reliable and reproducible results. Different reports suggest that low-quality RNA has a severe effect in pPCR quantification [58], transcript estimation [59], differential expression [60] and cDNA synthesis [61] that interferes with gene expression studies.

As it has been discussed above, to obtain high-quality RNA represents a fundamental step for high technology platforms including NGS that have provided many valuable insights into biological systems.

The modified protocol presented in this study demonstrated an increase in RNA yield and quality from all the carbon sources (Fig 2c and Table 1) compared to commercially available kits, which have been reported as the first option for Gram-negative bacteria, because they are rapid, capable of high-throughput analysis and cost-effective [35,62,63]. The use of RNAzol^®^ RT as a single step procedure removed DNA contamination without DNase treatment and reduced RNA time handling and helped diminished sample degradation, as has been tested in similar methods [63].

Regarding the cells grown on 2,4-TDA, pooling was an important key in the modified method. Pooling samples may provide a solution when RNA input is insufficient in a single sample for subsequent analyses [64]. A previous report showed that RNA isolated from pooled *Gyrodactylus salaris* samples was useful for increasing total RNA quality and yield [65]. Moreover, the use of pooling biological samples has been tested for the detection of gene expression changes via microarray [66]. Considering the low RNA or DNA inputs (ng/μL) which cannot be efficiently precipitated, the use of a carrier material has been studied as an effective alternative in some protocols [67]. Glycogen is regularly used in several molecular biology applications precipitating nucleic acids in solution to improve the formation of a visible pellet that simplifies downstream sample processing [67,68]. Finally, the use of a purification and concentration step is highly recommended for removing any phenol trace in RNA extraction involving guanidine-phenol based reagents as RNAzol^®^ RT [69]. This final step has been reported previously in other RNA extraction protocols due to it ensures the recovery of highly concentrated and pure RNA that be used for downstream applications afterwards [70–72].

## Conclusions

In the present study, five different methods for RNA isolation from *Pseudomonas capeferrum* TDA1 grown on succinate, phenol and 2,4-TDA were compared. Conventional methods failed to yield high quality RNA from cells grown on 2,4-TDA (Table 1). Therefore, a modified RNAzol RT protocol was developed and demonstrated to be the most efficient to obtain high-quality total RNA from 2,4-TDA grown cells. The modified RNAzol RT method tackles the problem of RNA degradation, its interaction with phenolic compounds and the removal of organic contaminants effectively. Furthermore, the protocol showed to yield high quality RNA for cells grown on phenol, another aromatic carbon source as well as cells grown on succinate. In fact, all bacteria known to aerobically degrade complex aromatic compounds use the same machinery of oxygenation enzymes that release metabolic degradation by-products known to interfere with RNA. Therefore, we are convinced that the present protocol can be used as a guideline as a guideline to improve total RNA extraction from all bacterial on samples from all bacterial cultures growing on complex aromatic carbon sources.

## Supporting information

S1 Fig Bioanalyzer results of total RNA isolated from *P*. *capeferrum* TDA1 grown on 2–4 TDA using the RNeasy method. (TIF)

S2 Fig Bioanalyzer results of total RNA isolated from *P*. *capeferrum* TDA1 grown on 2–4 TDA using the RNeasy power plant method. (TIF)

S3 Fig Bioanalyzer results of total RNA isolated from *P*. *capeferrum* TDA1 grown on 2–4 TDA using the phenol/chloroform method. (TIF)

S4 Fig Bioanalyzer results of total RNA isolated from *P*. *capeferrum* TDA1 grown on 2–4 TDA using the TriFast method. (TIF)
