# Supplementary figures and images for: An optimized method for RNA extraction from the polyurethane oligomer degrading strain Pseudomonas capeferrum TDA1 growing on aromatic substrates such as phenol and 2,4-diaminotoluene
Source: PLoS One. 2021 Nov 15;16(11):e0260002. doi: 10.1371/journal.pone.0260002 (PMC8592408; doi:10.1371/journal.pone.0260002)

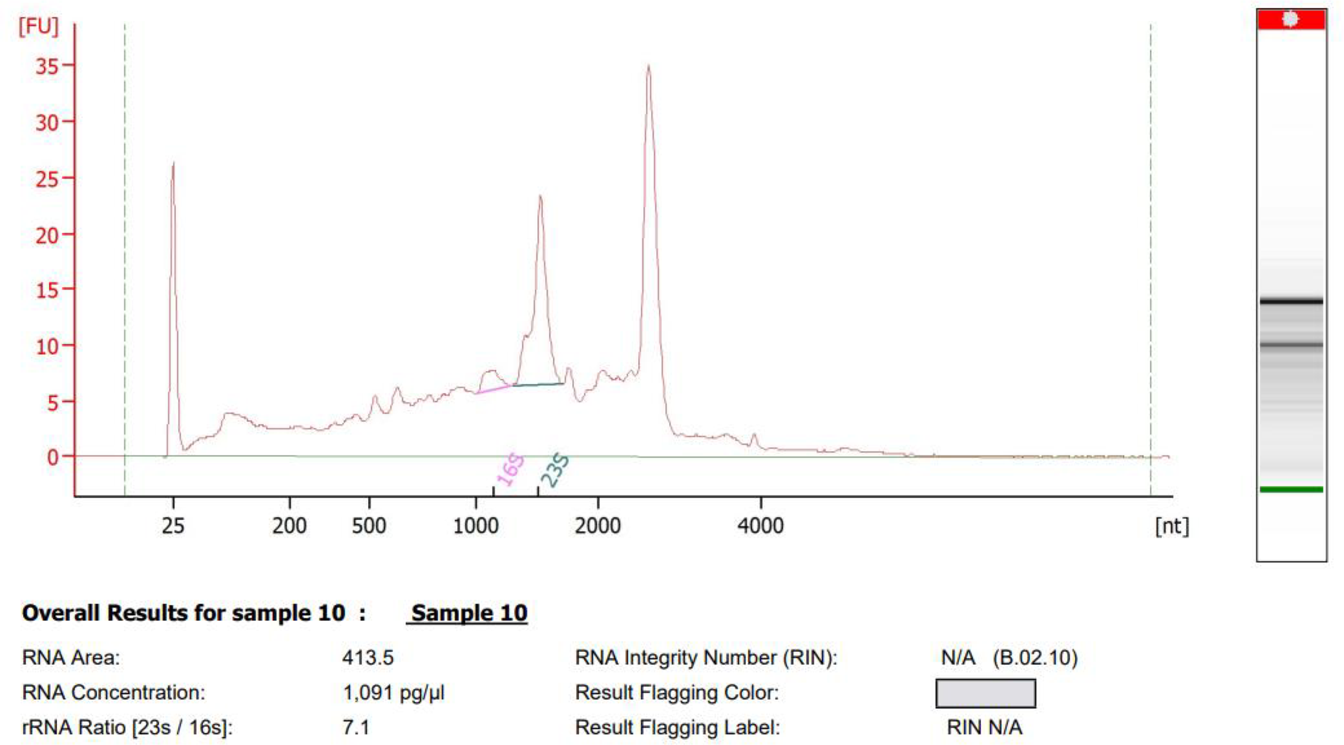

Supplement: S1 Fig — (TIF) [file pone.0260002.s001.tif]

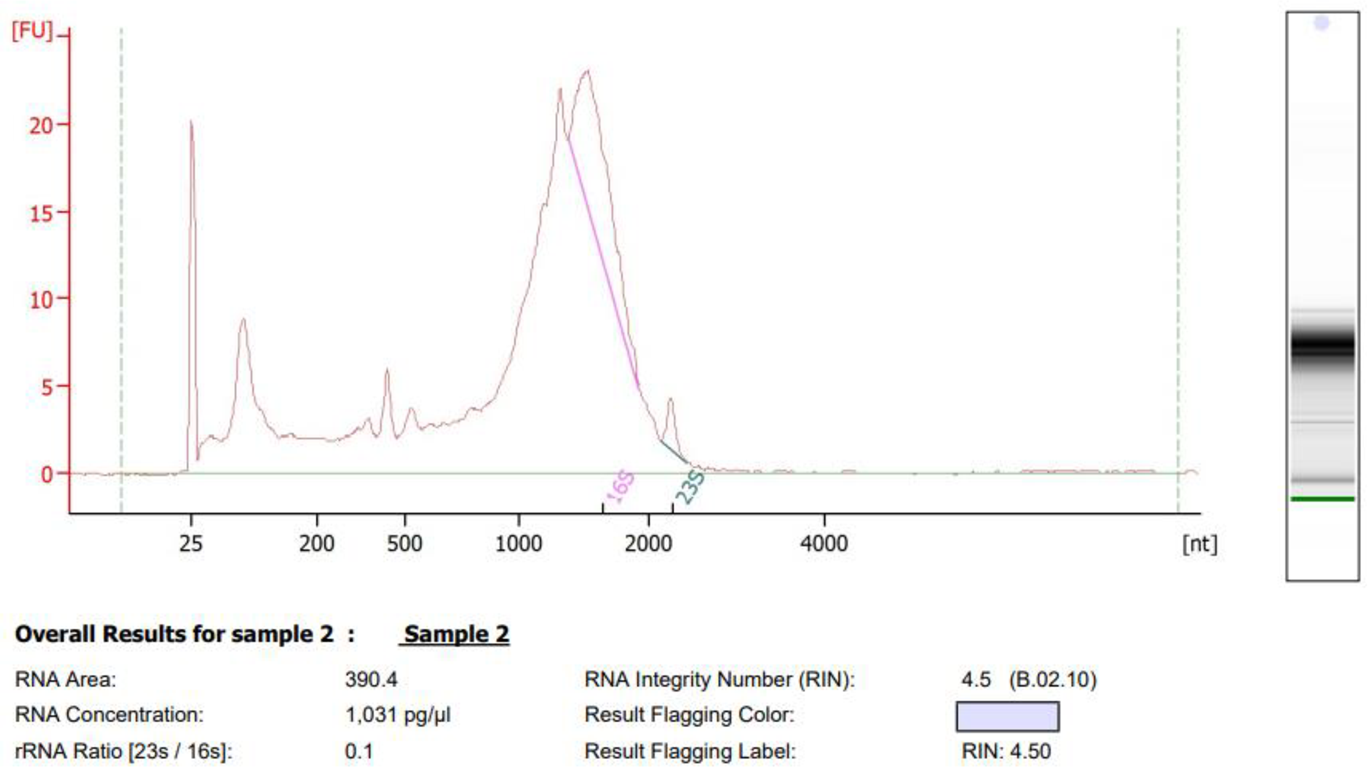

Supplement: S2 Fig — (TIF) [file pone.0260002.s002.tif]

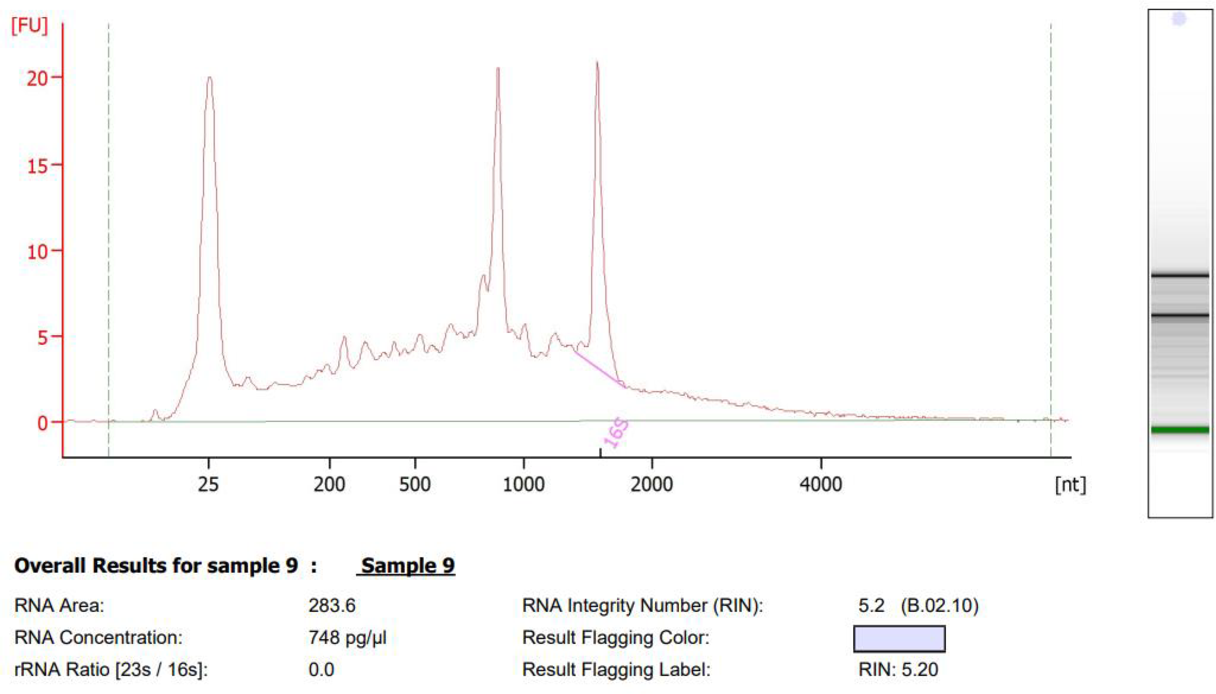

Supplement: S3 Fig — (TIF) [file pone.0260002.s003.tif]

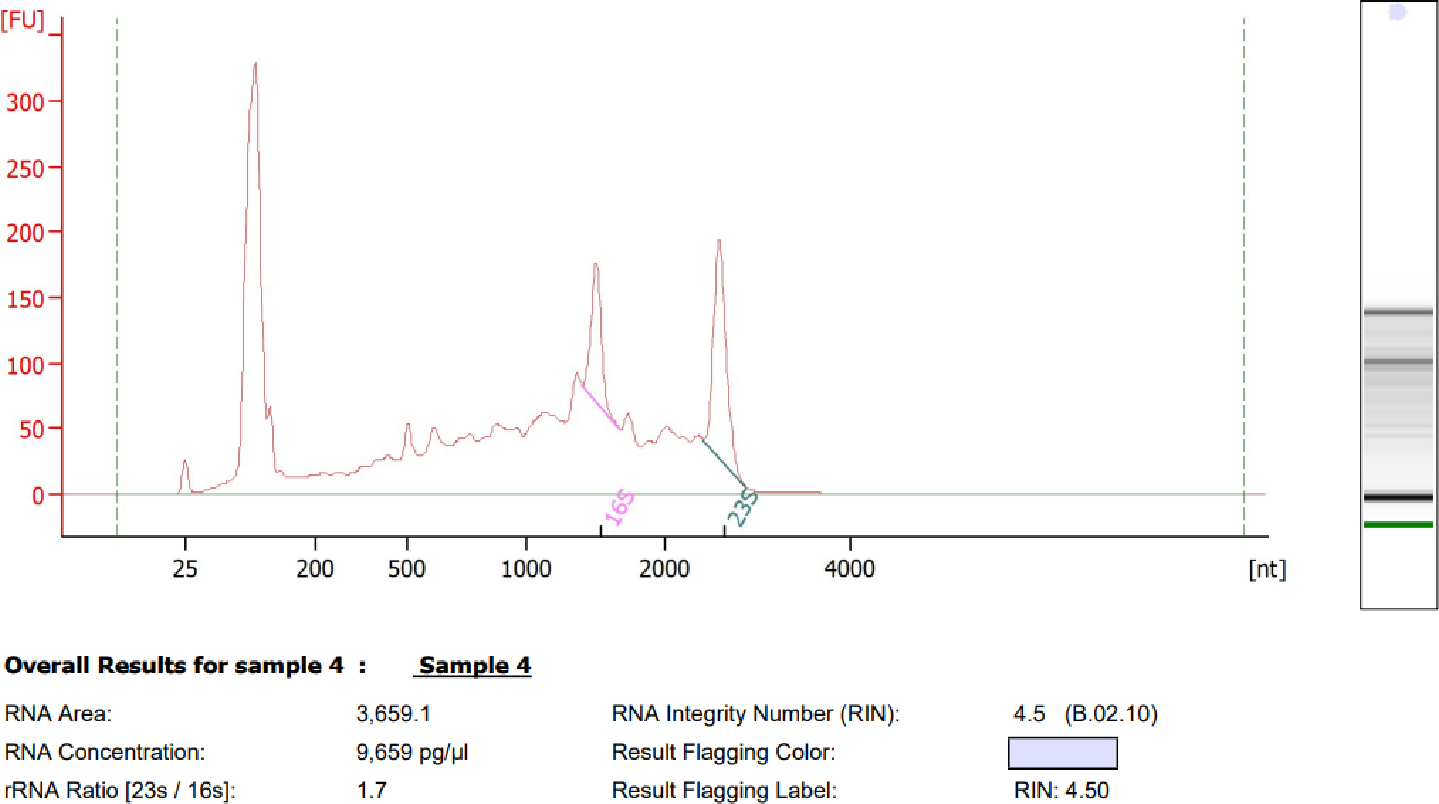

Supplement: S4 Fig — (TIF) [file pone.0260002.s004.tif]
